# Anterior pituitary gland T1 signal intensity is influenced by time delay after injection of gadodiamide

**DOI:** 10.1038/s41598-020-71981-0

**Published:** 2020-09-11

**Authors:** Carlo A. Mallio, Laura Messina, Marco Parillo, Gianguido Lo Vullo, Bruno Beomonte Zobel, Paul M. Parizel, Carlo C. Quattrocchi

**Affiliations:** 1 Unit of Diagnostic Imaging and Interventional Radiology, Departmental Faculty of Medicine and Surgery, Università Campus Bio-Medico di Roma, Via Alvaro del Portillo, 21, 00128 Rome, Italy; 2 Department of Radiology, Antwerp University Hospital, Edegem, Belgium

**Keywords:** Health care, Medical research

## Abstract

To test the hypothesis of washout from the anterior pituitary (AP) gland after serial injections of gadodiamide. We included 59 patients with history of at least 5 injections of gadodiamide. Values of mean signal intensity of the AP and of the central pons were measured on unenhanced sagittal T1-weighted images. AP-to-pons signal intensity ratios were calculated dividing the values of the AP by those of the pons. The measurements were performed using MR images acquired at four different time points including baseline (prior to any gadodiamide injection), minimum post-injection time delay, maximum post-injection time delay, and last available MR scans. Normalized ratios (i.e. ratios divided total volume of injected gadodiamide) were also calculated. To assess the difference between ratios, non-parametric Wilcoxon signed-rank test was applied. The correlations were tested with non-parametric Spearman correlation coefficient. A p-value < 0.05 was considered as statistically significant. A statistically significant increase of AP signal intensity was found by comparing the baseline scans with both the minimum time delay (p = 0.003) and maximum time delay scans (p = 0.005). We found significant higher normalized ratios for minimum post-injection time delay with respect to maximum post-injection time delay (p < 0.001). The normalized ratios demonstrated a statistically significant negative correlation with the post-injection time delay (r = − 0.31; p = 0.006). The findings of this study suggest that washout phenomena of retained/deposited gadolinium from the AP are influenced by the total injected volume and post-injection time delay.

## Introduction

The pituitary gland plays a key role in regulating body homeostasis by integrating external and paracrine/autocrine pathways^1^. It is located into a small bony cavity called sella turcica and it has two anatomically and functionally distinct lobes, the anterior pituitary (AP) (or adenohypophysis) and the posterior pituitary (or neurohypophysis)^1^.

Gadolinium is a heavy metal with strong paramagnetic properties that is administered to humans during contrast-enhanced MR imaging studies in the form of gadolinium-based contrast agents (GBCAs)^2,3^.

Since 2014, T1 hyperintensity on unenhanced MRI images of some intracranial structures, including the dentate nucleus (DN) and the globus pallidus (GP), has been associated to multiple injections of GBCAs^4–12^. This association was very strong for those GBCAs with a linear structure, that are known to have a lower thermodynamic and kinetic stability as compared to those with a macrocyclic structure^13,14^.

The currently available scientific literature failed to demonstrate causal relationship between gadolinium deposition and clinical neurological and neuropsychological or MR imaging changes indicative of tissue disruption^15–21^. Moreover, preclinical animal as well as human autopsy studies showed no histological tissue changes associated to gadolinium deposition^6,22,23^.

Recently, it has been reported an increased signal intensity of the AP on unenhanced T1-weighted images in patients who had undergone intravenous injections of gadodiamide, suggesting gadolinium deposition or long-term retention^24^. After that, the presence of gadolinium within the AP has not been explored further by histochemical analysis to date.

Moreover, it has not been investigated whether the T1 signal changes observed with MRI in vivo represent permanent deposition or long-term retention and whether, taking into account the total volume of GBCA injected, a drop of AP signal intensity can be detected after a certain amount of time.

Herein, we test the hypothesis that washout mechanisms from the AP gland occur after serial injections of gadodiamide and are a function of the cumulative injected volume and the post-injection time delay.

## Materials and methods

This retrospective observational study was performed in accordance with the Declaration of Helsinki and was approved by the Institutional Ethical Committee of the University Campus Bio-Medico di Roma. The written informed consent for this specific study was waived by the aforementioned Committee. Based on our institutional policy, all patients signed the general agreement form to give their availability to the use of clinical and imaging data for research purposes.

### Subjects

We screened our in-house image database and selected a time span from January 2008 to June 2017. We identified a group of 59 consecutive patients who had undergone at least 5 intravenous GBCAs injections, each exclusively with 0.1 mmol/kg body weight of Omniscan, GE Healthcare (gadodiamide 0.5 mmol/ mL).

The exclusion criteria were: (1) empty sella; (2) previous neurosurgery; (3) abnormal renal function evaluated by calculating (Modification of Diet in Renal Disease study equation) the estimated glomerular filtration rate (GFR) from a blood sample obtained less than 3 months prior to the inclusion (GFR < 60 mL/min); (4) unsatisfactory images due to MR artifacts or absence of unenhanced sagittal T1 images; (5) presence of neoplastic lesions involving the pituitary gland.

### MR imaging protocol

Imaging data were acquired at 1.5-T MRI system (MAGNETOM Avanto B13; Siemens, Erlangen, Germany), configured with a 12-element designed head matrix coil. The in-house MRI protocol includes the following sequences as previously described^24^: axial FLAIR, coronal TSE T2- weighted with and without fat suppression, and sagittal two-dimensional (2D) TSE T1 weighted (TR 503 ms, TE 11 ms, matrix 256 × 256, FOV 25 cm^2^, slice thickness 4 mm), conducted before and after intravenous administration of gadodiamide.

Based on the retrospective study design, small variations of MR parameters were allowed.

### Image data analysis

The quantitative analysis of the relative signal intensity of the AP gland was conducted using a previously reported method^24^.

Using a Carestream PACS workstation, the signal intensity of 5 random voxels was sampled in the AP on the mid-sagittal slice (including the pituitary stalk) of the unenhanced sagittal T1 images.

The mean signal intensity of the 5 voxels was taken as mean signal intensity of the AP. On the same slice an oval ROI of the central pons containing values of mean signal intensity was drawn.

Then, signal intensity ratios were calculated dividing the values of the AP by those of the pons obtaining AP-to-pons ratios.

All the measurements were performed at 4 timepoints: (1) at the first unenhanced MRI scan before any administration of gadodiamide (Baseline_Ratio); (2) at the MRI performed with the minimum post-injection time delay after the previous gadodiamide injection (Min_Delay_Ratio); (2) at the MRI performed with the maximum post-injection time delay after the previous gadodiamide injection (Max_Delay_Ratio); at the last available MRI scan for each subject (Last_Ratio). The last available brain MRI scan for each subject was the one performed at the greatest amount of previous gadodiamide cumulative doses until June 2017.

The ratios were compared between the first (Baseline_Ratio) and each individual timepoint (Min_Delay_Ratio, Max_Delay_Ratio and Last_Ratio) for all the subjects.

To correct for the different dose of injected gadodiamide at each timepoint and better clarify the potential effect of both time delay and total cumulative injection volume, we further divided the ratios obtained at the minimum and maximum post-injection time delay by the total volume of injected gadodiamide, to normalize the ratios (Norm_Min_Delay_Ratio and Norm_Max_Delay_Ratio), and adjust for the effect of the cumulative doses.

All the ROI drawings and image visual inspections were performed by consensus of two experienced neuroradiologists (C. C. Q., 16 years of experience; C. A. M., 9 years of experience), who were blinded to any patients’ information.

### Statistical analysis

Descriptive statistics, means, ranges and standard deviations were assessed to understand central tendencies in each group. The normality of data distribution was tested using the Kolmogorov–Smirnov test. The differences between different ratios, obtained at variable timepoints, were explored by means of non-parametric Wilcoxon signed-rank test (2-sided p-value within 99% confidence interval).

The correlations were tested with non-parametric Spearman correlation coefficient.

Analyses were conducted by using the Statistical Package for the Social Sciences software package (version 24.0), setting the a priori significance level at p < 0.05.

## Results

Twenty-two patients were excluded from the analysis: 18 were affected by motion artifacts or pre-contrast sagittal T1-weighted images were missing, 3 were diagnosed with empty sella and 1 showed a metastatic lesion centered in the pituitary gland. Therefore, a group of 37 patients (15 males and 22 females) was finally considered for analysis.

Clinical indications to gadolinium-enhanced brain MRI for the patients was as follows: meningioma (n = 7), optic neuritis (n = 1), brain metastases (n = 14), glioblastoma (n = 1), multiple sclerosis (n = 10), central nervous system lupus (n = 1), arteriovenous malformation (n = 1), hypertensive encephalopathy (n = 1) and leukoencephalopathy (n = 1).

Descriptive data of the variables collected for each patient and each timepoint are provided in Table 1.

**Table 1:** Descriptive data of the included patients.

	Minimum	Maximum	Mean (± standard deviation)
Age (years)	23	86	53 (14.68)
Minimum delay (days)	1	376	93 (87.93)
Maximum delay (days)	103	810	373 (208.89)
Last (days)	39	700	288 (191.59)
Doses minimum delay (N)	1	19	3.64 (3.57)
Doses maximum delay (N)	1	18	6.21 (3.72)
Doses last (N)	5	19	8.32 (3.80)
Volume minimum delay (mL)	10	266	51.08 (50.06)
Volume maximum delay (mL)	10	252	87.02 (52.08)
Baseline_Ratio	0.75	1.10	0.99 (0.07)
Min_Delay_Ratio	0.81	1.29	1.03 (0.09)
Max_Delay_Ratio	0.81	1.18	1.02 (0.09)
Last_Ratio	0.90	1.17	1.05 (0.06)
Norm_Min_Delay_Ratio	0	0.09	0.038 (0.027)
Norm_Max_Delay_Ratio	0	0.07	0.017 (0.014)

The last MRI is intended as the one obtained after the greatest amount of previous gadodiamide cumulative doses. The maximum and minimum time delay MRIs are defined as those obtained respectively after the maximum and the minimum time lapse since the last gadodiamide injection regardless the amount of previous gadodiamide cumulative doses.

As expected, there was a significant increase of AP T1 signal intensity in the Last_Ratio with respect to the Baseline_Ratio (p < 0.001). A statistically significant result, albeit less strong, was also obtained with Min_Delay_Ratio (p = 0.003) and Max_Delay_Ratio (p = 0.005).

**Figure 1:**
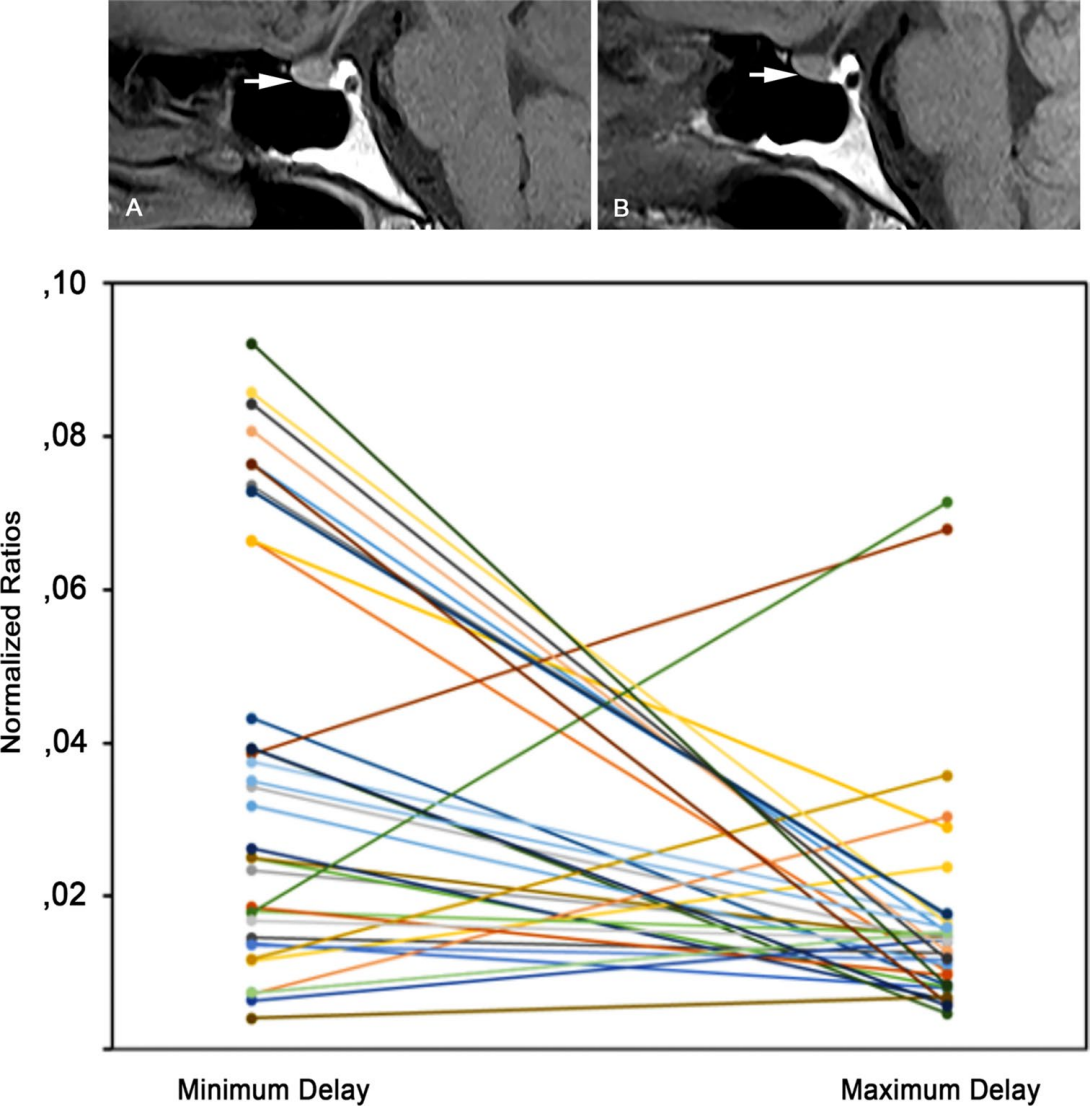
Sagittal unenhanced T1 MR images acquired after one previous gadodiamide injection with post-injection time delay of 1 day **(A)** and after four previous gadodiamide injections with post-injection time delay of 180 days **(B)**. A slightly greater signal intensity of the anterior pituitary gland is visible in **(A)** with respect to **(B)** (arrows). The graph of the lower panel depicts a trend towards reduction of normalized ratios from minimum post-injection delay to maximum post-injection time delay. Each line represents one individual patient.

The comparison between Min_Delay_Ratio and Max_Delay_Ratio revealed absence of significant differences (p = 0.55). However, by comparing normalized ratios, we found significantly higher values of Norm_Min_Delay_Ratio than Norm_Max_Delay_Ratio (p < 0.001) (Fig. 1).

**Figure 2:**
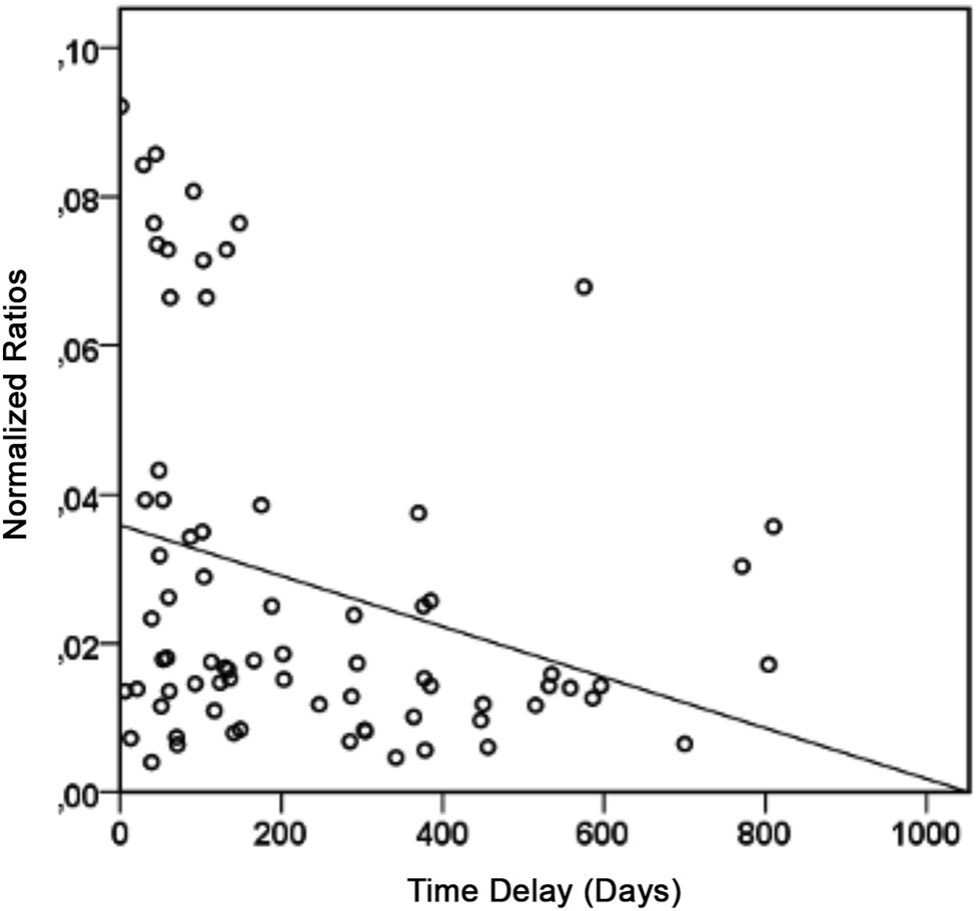
Scatterplot showing the significant negative association between normalized ratios and post-injection time delays.

Moreover, all the normalized ratios taken together (Norm_Min_Delay_Ratio and Norm_Max_Delay_Ratio) demonstrated a statistically significant negative correlation with the post-injection time delay (r = − 0.31; p = 0.006) (Fig. 2).

## Discussion

In this study, we found an increase of the signal intensity of AP on unenhanced T1 weighted MR images obtained at minimum as well as maximum post-injection time delay, in patients with normal renal function exposed to serial gadodiamide injections. The result confirms a previous report showing increased T1 signal intensity of the AP after chronic exposure to GBCAs^24^.

The values calculated at minimum post-injection delay were not different than those obtained at maximum post-injection time delays. However, it should be taken into consideration that patients usually undergo MR imaging at shorter post-injection time delays early after the diagnosis of their disease and tend to have lower cumulative doses received, whereas the patients with longer post-injection time delays are those undergoing follow-up studies and, as long survivors, tend to have higher cumulative doses of gadolinium. Thus, to discriminate the effects of these two major variables potentially affecting the results (i.e. post-injection time delay and the cumulative volume of previous injected GBCAs), we normalized the signal intensity ratios by the volume of gadodiamide administered at the time of the measured ratio. Interestingly, we found a statistically significant difference between normalized ratios, as images obtained at minimum post-injection time delay showed higher signal intensity than those obtained at maximum post-injection delays and despite a lower cumulative injected volume of gadodiamide. This finding suggests that at least partial washout phenomena of retained/deposited gadolinium from the AP are influenced by the total injected volume and post-injection time delays. The significant negative correlation between normalized ratios and post-injection time delay reinforces this concept.

It is known that gadolinium colocalizes with other elements in tissues^25–27^. Indeed, a colocalization between gadolinium and iron has been recently pointed out in some brain structures including DN and GP^25–27^.

In patients with transfusion-related iron overload, such those with β-thalassemia major or myelodysplasia, circulating iron first deposit in the reticuloendothelial system and subsequently in several parenchymal organs^28,29^. In such cases, the intracranial structures more prone to iron deposition are those without blood–brain barrier such as pineal gland, choroid plexus, and pituitary gland^28,29^. Thus, it is to be considered possible that gadolinium and iron might also colocalize within the AP.

The embryogenesis of the pituitary gland is complex. It originates from the joint of two distinct parts, the Rathke’s pouch, which is formed as a dorsal evagination of the stomodeum, and the infundibulum, arising as a ventral extension of the diencephalon^30,31^. With progression of the pituitary development, the cells of the anterior wall of Rathke’s pouch proliferate fast in order to form the adenohypophysis. The growth of these cells produces two compartments (Atwell's fossae) that will be separated by a cellular median septum and initially filled with mesenchymal elements. The subsequent migration of mesenchymal elements from the fossae Atwell's fossae to the anterior surface of the infundibulum will eventually give rise to the blood vessels of the hypophyseal portal system^30,32^. Of note, the AP is known to be the most vascularized among of all mammalian tissues, reached by about 0.8 mL/g/min of blood from the portal system^30,33^. Moreover, the histology of the AP in characterized by interlacing cords of large polygonal cells separated by an extensive network of sinusoidal capillaries with fenestrated endothelium, in order to promote uptake of the secreted hormones^30,34^.

Therefore, the rich vascular supply as well as the absence of blood–brain barrier and the fenestrated endothelium of sinusoidal capillaries make the pituitary gland a perfect site for a fast wash in and fast washout of elements traveling through the bloodstream.

Gadolinium deposition in tissues has been investigated by several groups in preclinical studies with mass spectrometry, histopathology and MR imaging but nobody focused on the AP to date^21,35–37^.

Thus, the presence of gadolinium deposited within the AP has not been confirmed yet. On this respect, our results point towards gadolinium washout from the AP after a certain amount of time delay, suggesting retention rather than deposition; that is in keeping with the gross anatomy and histology of the AP. Since we found a significant increase of signal intensity on unenhanced T1 weighted images acquired at minimum as well as maximum post-injection time delays, a contribution of gadolinium deposition occurring at cellular and extra-cellular level within the AP cannot be ruled out.

Currently, the most accepted mechanism of gadolinium deposition after the i.v. injection of GBCA with linear structure such as gadodiamide is based on the entry into the brain through the cerebrospinal fluid and the glymphatic pathway and on the transmetallation with other elements showing high affinity for gadolinium-binding chelators^38–41^. Therefore, the rich vascular supply of the AP might also not be critical in this context.

Indeed, the results reported in this study need to be further investigated and validated with analytical techniques on pre-clinical models.

This study has limitations that should be taken into account and might affect a generalization of the results. First, due to the retrospective nature of the study, we allowed small variations of the parameters of the standard MR imaging protocol. Second, the measurement of the AP signal intensity was performed on a single-slice sagittal T1-weighted 4 mm image; such a measurement is extremely difficult, is not performed on the entire volume of the AP and is greatly affected by images artifacts even if small. Third, the high T1 signal intensity is non-specific and is not restricted to gadolinium but occurs with many other paramagnetic substances. Fourth, it was not possible to consistently retrieve laboratory data about hormonal status of the patients.

## Conclusions

We found a significant increase of T1 signal intensity on images obtained at minimum post-injection time delay as compared to those at maximum post-injection time delay. This result and the negative correlation between normalized ratios and post-injection time delays suggest at least partial washout phenomena of retained/deposited gadolinium from the AP that are influenced by the total injected volume and post-injection time delay.
